# Can a Knee Brace Prevent ACL Reinjury: A Systematic Review

**DOI:** 10.3390/ijerph18147611

**Published:** 2021-07-17

**Authors:** Bianca Marois, Xue Wei Tan, Thierry Pauyo, Philippe Dodin, Laurent Ballaz, Marie-Lyne Nault

**Affiliations:** 1 CHU Ste-Justine, 7905-3175 Côte Ste-Catherine, Montréal, QC H3T 1C5, Canada; marois.bianca@gmail.com (B.M.); philippe.dodin.hsj@ssss.gouv.qc.ca (P.D.); laurent.ballaz@gmail.com (L.B.); 2 Department of Surgery, Université de Montréal, 2900 boul. Edouard-Montpetit, Montreal, QC H3T 1J4, Canada; xue.wei.tan@umontreal.ca; 3 Shriners Hospital, McGill University Health Center, 1003 Decarie Blvd, Montreal, QC H4A 0A9, Canada; Tpauyo@gmail.com

**Keywords:** knee brace, incidence rate, anterior cruciate ligament reconstruction, second injury, re-tear

## Abstract

This systematic review aimed to investigate whether the use of a knee brace when returning to sport (RTS) could prevent a second injury after anterior cruciate ligament reconstruction (ACLR). This study was registered with the PROSPERO database and followed PRISMA guidelines. A systematic search of PubMed, Ovid Medline, Ovid All EBM Reviews, Ovid Embase, EBSCO Sportdiscus and ISI Web of Science databases for meta-analysis, randomized controlled trials and prospective cohort studies published before July 2020 was undertaken. The inclusion criteria were: (1) Comparing with and without a brace at RTS, (2) follow up of at least 18 months after ACLR, (3) reinjury rates included in the outcomes. Two reviewers independently extracted the data. Quality appraisal analyses were performed for each study using the Cochrane Collaboration tools for randomized and nonrandomized trials. A total of 1196 patients in three studies were included. One study showed a lower rate of reinjury when wearing a knee brace at RTS. One study found the knee brace to have a significant protective effect for younger patients (≤17 years). The effectiveness of knee bracing when RTS remains ambiguous. Current data cannot support that using a knee brace when RTS will decrease the rate of reinjury after ACL reconstruction.

## 1. Introduction 

More than 200,000 anterior cruciate ligament (ACL) injuries occur in the United States every year [1,2,3], with over 100,000 individuals undergoing ACL reconstruction (ACLR) annually. Rehabilitation plays a crucial role in the success of the primary reconstruction; nevertheless, subsequent knee injury is common following ACLR. Recent literature shows a rate of ACL retear after ACLR when returning to sport between 8% and 23%, depending on the population and clinical implications [4,5]. The risk of a second injury is higher in patients who (1) return to cutting and pivoting sports, (2) do not meet the return to sport criteria before returning to sport, and (3) returning to pivoting sports earlier than 9 months after ACLR [6,7]. A second ACL injury, either a graft rupture or contralateral ACL injury after ACLR, negatively impacts knee function [8,9,10], quality of life [11,12], accelerates degenerative changes in the knee [11,13,14,15] and challenges an athlete’s career [16,17,18,19]. The long rehabilitation period, difficulty in returning to the pre-injury level and fear of reinjury may also impact the athlete’s career when returning to sport [20,21]. Biomechanical studies have shown that wearing a functional brace after ACL injury provides the ACL-deficient or ACL-reconstructed knee with near normal stability [22,23,24,25,26]. The knee brace is specifically used to normalize tibiofemoral joint mechanics and to protect the graft by preventing abnormal anterior tibial translation (ATT), excessive strain and elongation of the ACL graft [27]. A recent survey managed by the American Orthopaedic Society for Sports Medicine demonstrated that 63% of surgeons who perform ACLR recommend the use of functional braces when retuning to sport [28]. However, the effectiveness of bracing to reduce reinjury rates when returning to sport following ACLR is controversial. 

The purpose of this systematic review was to present current findings on the effect of wearing a knee brace on preventing ACL reinjury after return to sport (RTS) in ACLR patients. The primary aim was to evaluate the difference in reinjury risk between patients with and without a knee brace when returning to sport. The secondary aim was to examine the impact of the knee brace on overall knee function, symptoms, sports activity, and quality-of-life using questionnaires (International Knee Documentation Committee (IKDC) score, Lysholm, Tegner, etc.) and on compliance to the intervention program. 

## 2. Materials and Methods

This systematic review was performed according to the Preferred Reporting Items for Systematic Reviews and Meta-Analysis guidelines [29] (PRISMA). This study was registered with the PROSPERO database on 5 July 2020 under the following question, «Does the use of knee brace, after ACLR, when returning to sport reduce the rate of second injury?». Studies evaluating the effects of wearing a knee brace when returning to sport after ACLR compared to a control group (unbraced) were considered in this systematic review. 

### 2.1. Search Strategy

A detailed search strategy was developed with the chief institutional librarian and is presented in Appendix A. The search was performed in six electronic databases (PubMed, Embase, Sportdiscus, Medline, Web of Science and EBM reviews) and included studies published before July 2020. A combination of the following keywords was used: ACL, reconstruction, orthotics and second rupture. There were no further requirements for the studies, and grey literature was included in the initial database search. The references of eligible studies were also assessed to determine whether additional publications could be included in this review. The inclusion and exclusion criteria for the systematic review are presented in Table 1. 

### 2.2. Study Selection

From the original search, duplicates were removed and articles were individually screened by title/abstract by two reviewers (BM and XWT). When the two reviewers disagreed during the screening process, a third reviewer (MLN) made the final decision. The full-text articles of the included studies were reviewed. The articles that did not meet the inclusion criteria were excluded. The remaining studies were included in this review. The reference lists of excluded articles from the full-text screening as well as those of included articles were reviewed for potentially eligible articles that may have been missed in the electronic database search.

Data were extracted by two independent reviewers (BM and XWT), and disagreements were resolved by consensus (BM, XWT and MLN). Key variables regarding the patient’s demographics, graft type, follow-up time after surgery, overall knee function, sports activity and compliance to the intervention program were extracted. Quality-of-life questionnaire results (International Knee Documentation Committee (IKDC) score, Lysholm, Tegner, etc.) and findings (such as reinjury rates) were extracted.

Two reviewers (BM and XWT) independently assessed the quality of the reviewed studies by identifying factors that could influence the interpretation of the results. If the two reviewers did not agree, a third reviewer (MLN) made the final decision. The reviewers were not blinded to authors, journal or publication. The quality of all articles was performed with the Cochrane Risk of Bias Tools for Randomized and Nonrandomized Clinical Studies [30,31]. The risk of bias assessment for randomized controlled trials (RCT)’s used 6 criteria and 7 criteria for the nonrandomized clinical studies.

## 3. Results 

**Figure 1:**
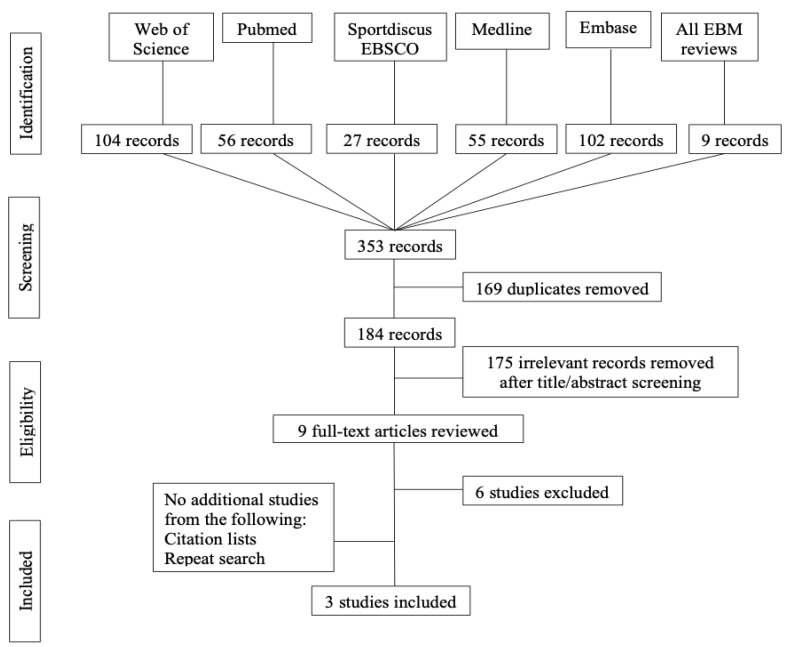
PRISMA flow chart.

The PRISMA flow chart for this systematic review is presented in Figure 1. A total of three studies were included in the present review: 1 RTC [32] and 2 prospective cohort studies [2,33].

### 3.1. Risk of bias assessment

The results of the risk of bias evaluation for each study included within the final analysis are presented in Table 2 and Table 3. Three articles included in this systematic review had an intermediate risk of bias. 

### 3.2. Study Results

The details of the included studies are presented in Table 4. A total of 1213 participants in three level II studies (one randomized study design, two prospective cohort studies) [2,32,33] with a follow-up fluctuating from 24 to 96 months were included. No additional studies were found after a manual search of the reference lists. Six articles were excluded in the full-text revision (four did not compare two different groups, and two focused on postoperative braces).

Perrone et al. [2] published a retrospective review comparing the rate of reinjury following ACLR with hamstring autograft of 219 braced patients to a historical control cohort of 140 unbraced patients. The braced cohort was prescribed a knee brace to wear when returning to cutting and pivoting sports for a minimum of 2 years. The type of knee brace they used was not available. Rehabilitation was similar for both cohorts and return to sport was permitted after gradually increasing training intensity 6 months postsurgery. The follow-up rate of the braced cohort 3 years after surgery was 65%. The authors found significant differences in the reinjury rates between both cohorts. Overall, 14 patients (10%) suffered graft injury in the braced cohort compared to 29 patients (21%) in the unbraced cohort (*p* = 0.028). Early graft injury was higher in the unbraced cohort (12 patients (9%) within the first year after surgery compared to the braced cohort which had 2 patients (1%) (*p* = 0.011)). No significant differences were found between the groups for contralateral limb injury. 

McDevitt et al. [32] randomized one hundred patients via coin toss, immediately after surgery, into braced and unbraced groups. They aimed to understand the effect of a knee brace when returning to sport with subjective and objective outcomes, including reinjury rates between both groups. The braced group wore an off-the-shelf functional knee brace for all cutting, pivoting and jumping activities, for a minimum of 1 year following surgery. Pivot shift test, Lachman test, functional knee testing, International Knee Documentation Committee evaluation form score (IKDC) [34], Lysholm score, KT-1000 arthrometer examination and isokinetic testing were assessed at a minimum of 2 years after the surgery. There was no difference between the groups at the 2-year follow-up in any of the measured outcomes. A total of two braced (4%) and three unbraced participants (6%) suffered reinjury. They did not see any significant difference between the unbraced and braced cohort in reinjury and complication rates after ACLR (*p* > 0.05). 

The MARS group [33] studied the rehabilitation predictors of 2-year outcomes in 843 patients who underwent revision ACLR surgery. At the time of the surgery, the 83 physicians participating in the study answered « yes » or « no » to questions regarding their knee brace prescription and each patient’s use of it. A total of 253 patients (30%) were prescribed an ACL brace for return to sport and 573 patients (68%) returned to sport without an ACL brace. Patients were assessed at baseline and at 2 years follow-up with a combined questionnaire that included the Knee Injury and Osteoarthritis Outcome Score (KOOS) [35], the International Knee Documentation Committee (IKDC) Subjective Knee Form [34] and the Marx activity rating scale [36]. The authors found that using an ACL knee brace for the return to sport was associated with a better score in the KOOS sport/recreation questionnaire at 2 years (*p* = 0.019). There was no significant difference between groups in the prevalence of graft failures; a total of 20 graft tears (3.5%) were reported from the unbraced group and 5 graft tears were reported from the braced group (2%) (*p* = 0.23). 

In all three studies, the outcome was compared between bracing and nonbracing groups following ACLR. All studies reported subjective outcomes including Lysholm, International Knee Documentation Committee (IKDC) [34], and Mark Activity Rating Scale [36]. McDevitt et al. [32] used bone–patellar tendon–bone autograph, Perrone et al. [2] used hamstring grafts and the MARS group [33] used both bone–patellar tendon–bone autograph and allograft. 

In all three studies, the inclusion and exclusion criteria were almost identical, except for the following: two studies excluded patients if they had previous surgery to the lower limb, associated meniscal tears or subsequent ligament tears, and those who had a prior ipsilateral or contralateral ACL reconstruction [32,33]; one study included only patients that underwent surgical reconstruction within 8 weeks of injury [32]; one study followed patients who underwent revision ACLR [33]. Among the three studies, two were randomized [2,32], while the MARS group [33] did not randomize the participants, leaving the choice of using a knee brace up to the physicians. 

### 3.3. Subsequent Injuries 

Graft rupture rates for each study are displayed in Table 5. Perrone et al. [2] found that 14 patients (10%) in their brace cohort had a graft failure as opposed to 29 graft failures (21%) in the control cohort (*p* < 0.05). In the brace cohort, out of those 14 graft failures, two (14%) occurred in the first postoperative year and five (36%) occurred within the first 2 years following the surgery. In the control cohort, 12 of the 29 reinjuries occurred within the first postoperative year. The early graft rate (within 1 year of surgery) was lower in the braced group (1%) than in the unbraced group (9%). Subgroup analysis indicated a higher rate of reinjury for the younger patients (i.e., 17 years and younger). The reinjury rate for the younger patients in the braced group was 2% (2 of 115), and it was 12% for the unbraced group (11 of 89). No statistically significant difference was observed in contralateral ACL injury rates after ACLR between the braced and unbraced cohorts. In the McDevitt et al. [32] study, a total of two braced and three unbraced participants were reinjured. One braced participant fell and fractured her patella while she wasn’t wearing a brace 6 weeks after surgery. The other participant in the braced group partially tore her ACL graft 11 months post-surgery while wearing her knee brace. In the unbraced group, the injuries were two graft tears (at 9 months and 1-year post-surgery) and one meniscus tear at 14 months after ACLR. These three injuries occurred while playing varsity sports. Subsequent injury factors were not detailed in the MARS group [33] study. 

### 3.4. Compliance

Two studies included data on brace use within the braced cohort [2,32]. Compliance and questions to determine if there were problems with the knee brace were assessed in the McDevitt et al. [32] study, with a questionnaire at final follow-up for the braced group. The average time from surgery to final follow-up was 29 months (range: 24–42 months). Participants in the braced group were prescribed a functional knee brace 6 weeks after surgery and asked to wear the brace daily for 6 months and for all rigorous activities for at least 1 year. They found that eight (21%) of their braced participants stopped wearing their functional brace before the prescribed 1-year period (mean of 8 months, range 6–10 months). Participants who stopped wearing the brace thought the brace was negatively affecting their sport performance. The other participants of the McDevitt et al. [32] braced cohort thought the brace gave them more confidence and they felt a greater sense of security (safer) with the brace. Perrone et al. [2] used a survey inspired by Webster et al. [10] and added questions on the duration of postoperative bracing use. For this study, all patients were prescribed a functional ACL brace to be used during participation in cutting and pivoting sports for a minimum of 2 years. In their braced cohort, only 104 patients (75%) wore the brace for the recommended time (more than 1 year following the surgery). Compliance with brace wear during return to sport was not assessed in the MARS group [33] study.

### 3.5. Return to Sport Level 

After ACLR, most athletes seek to reach their preinjury level of performance. To determine an athlete’s capacity to return to sport after ACLR there are a range of functional performance tests. The return to sport level was studied by two articles. Detailed criteria for clearance to RTS were described in only one of the included studies. Perrone et al. [2] used the following criteria: full range of motion, quadriceps strength, single-leg step down or squat. RTS was gradually encouraged from 6 months after the reconstruction. They found that 63% of the participants in the braced cohort reported having returned to a very strenuous level of activity, including jumping and pivoting. In the unbraced cohort, 88% of the patients reported having returned to a strenuous level of activity. RTS level was evaluated using the Marx activity level score. In the McDevitt et al. [32] study, a self-reported questionnaire showed that only one (1 of 95) participant, non-braced, did not return to the same level of activity. The MARS group [33] did not study return to sport rates or level.

### 3.6. Quality of Life Questionnaires

All three studies used questionnaires to assess overall knee function and sports activity. The MARS group [33] findings suggest that patients who are prescribed an ACL brace have a better KOOS sport/recreation score at 2 years follow-up. They found that the odds of having a higher KOOS sport/recreation score is greater by 50% in patients who were prescribed a functional brace for sport (*p* = 0.019) compared with patients who didn’t wear a knee brace when returning to sport. Domains in which the major differences were noted were not available. In the other studies [2,32] differences in questionnaire scores were not significant between the braced and unbraced cohorts. 

## 4. Discussion

The purpose of this systematic review was to investigate the effect of knee braces when returning to sport after ACLR on reinjury rates. Many studies investigated the effect of the knee brace to prevent reinjury, as it became very commonly prescribed by physicians for better postoperative knee outcomes [37,38,39,40,41,42]. However, the studies available to support the use of a knee brace when returning to sport have a limited quality of evidence.

Yang et al. [42] also conducted a systematic review that included studies with ACLR patients. Their focus was different in terms of intervention and outcomes; they assessed the use of the knee brace in the rehabilitation phase rather than when returning to sport. Their purpose was to evaluate the outcomes on the knee functional scores and knee stability evaluations. One of the studies in our review was consistent with the results reported by Yang et al. [42] showing that functional bracing after ACLR may have beneficial effects on preventing early graft tear for younger patients (≤17 years). This population is unique, due to its active lifestyle, its heterogeneous morphology and its particularly high rate of ACL reinjury [2,10,19], which can have a negative impact on quality of life. The adolescent population is more at risk of having an ACL graft failure. However, findings from McDevitt et al. [32] suggest that, in a young and active population, the use of a functional brace when returning to sport does not appear to influence clinical outcomes after ACLR. It is important to note that their population size limited their ability to detect a difference between groups (*p* > 0.05). Moreover, there are no details on power calculation and sample size in their article, making it more difficult to interpret the results obtained. These contradictory findings illustrate the lack of quality research to support Yang et al.’s theory [42], making additional research in this area warranted. 

Postoperative knee bracing after ACLR has been shown to be associated with some additional adverse effects to the operated knee. Di Miceli et al. [43]^.^ showed that the use of a brace and delayed weight bearing after ACLR have a negative impact on long-term functional outcomes, according to the subjective IKDC score. Although the studies included in our systematic review did not find a statistically significant difference in the subjective IKDC score, the significant difference found in the MARS study for the KOOS score may be explained by the improvement in quality of life after ACLR revision. Furthermore, Moller et al. [44] found that patients that are treated with braces were more likely to see increased compression on the soft tissues of the limb. Other drawbacks to wearing a knee brace have also been reported in Styf et al. [45], including potential thigh atrophy, loss of flexion range of motion and increased fatigability during sports. Altered muscle activation has been reported in previous studies by patients wearing a knee brace while returning to sport and may be a factor that increases reinjury rates. The possibility that a knee brace may increase the risk of reinjury cannot be ignored [46]. It is important to note, however, that knee braces were used in the previous studies as a treatment plan in the rehabilitation phase, not while returning to sport.

One study with ACLR revision was included in this systematic review. Although the MARS group [33] only included revisions of ACLR surgery, it still evaluated the effect of the orthosis on surgical outcomes, and we believe that it was important to be included in our review, especially since it is a homogeneous revision cohort. One limitation with ACLR revision patients is that they may be more cautious about returning to sport or not returning to sport at all. Therefore, this may have had an impact on the number of reinjuries compared to a primary reconstruction cohort, leading to an overestimation of the protective effect of the orthosis.

This systematic review has some limitations. First, a lack of quality studies in the literature on this topic meant that only three studies were included in this systematic review. A meta-analysis could not be performed due to the heterogeneity in the methodology between each study. The studies were different in terms of outcome definition (i.e., age, return to sport testing, training status, tendon graft, type of knee braces, patient history of knee injury, other lower-extremity injuries or prescription of knee brace use). Furthermore, the definition of return to play is not standardized in the literature. Therefore, the rehabilitation protocol and the return-to-sport criteria were not specific or not mentioned in the included studies. Although the neuromuscular training program is known to be mandatory to improve outcomes [21], several studies have shown that a higher level of activity is related to a higher risk of ACLR graft tear [47,48]. Thus, in the MARS group [33], participants were returning to sport after revision surgery, which may have led to more caution in their level of activity compared to primary ACLR. This may explain the lower reinjury rates compared to the other studies. Furthermore, each study had a different purpose for knee brace use. The MARS group [33] let the enrolled physicians choose the scheduling of knee brace use. Prescribing a knee brace from 6 months postsurgery (as opposed to prescribing a knee brace when returning to sport) may have an impact on the study’s outcomes. In all three included studies, the reinjury rates were not the main outcome studied. The heterogeneity of the studies in regard to the population’s level of activity, age, graft type and rehabilitation protocol prevented any conclusions being drawn regarding the effect of knee brace on reinjury rates when returning to sports. 

## 5. Conclusions

This systematic review reinforces the message that there is clinical uncertainty regarding prescribing functional knee braces when returning to sport. No trends indicating a protective effect of knee braces against retear after ACLR were reported in this review due to a limited amount of included studies and the heterogeneity in their methods and outcomes. Choosing to use a knee brace for RTS depends on patient or physician preference. However, given the limited evidence, physicians need to be cautious when prescribing their usage. There is, therefore, a need to conduct quality research for an improved understanding of their protective effect. Furthermore, rapidly developing innovations in knee braces could make them more efficient in terms of prevention and compliance.

## Figures and Tables

**Table 1:** Inclusion and exclusion criteria.

Inclusion	Exclusion
Comparison of two (2) groups of patients who underwent anterior cruciate ligament reconstruction; Comparison between patients with brace and patients without brace when returning to play; Follow-up of minimum 18 months Primary or secondary outcomes must include reinjury rates	Multiligament injury; Postoperative brace studied;

**Table 2:** Results of systematic quality appraisal for each randomized study using the cochrane collaboration tool for assessing risk of bias in randomized trials.

	Risk of Bias for Included Randomized Trials						
Study	Random Sequence Generation	Allocation Concealment	Blinding of Subject/Personnel	Blinding Outcomes Assessment	Attrition	Selective Reporting	Overall
McDevitt et al., 2004	Intermediate	Low	Intermediate	Intermediate	Low	Low	Intermediate

**Table 3:** Results of systematic quality appraisal for each nonrandomized study using the cochrane risk of bias assessment tool for nonrandomized Studies of intervention.

	Risk of Bias for Included Nonrandomized Trials							
Study	Confounding	Subject Selection	Intervention Measurement	Departure From Intended Intervention	Attrition	Outcomes Measureent	Selective Reporting	Overall
Perrone et al., 2019	Intermediate	Low	Low	Low	Low	Low	Low	Intermediate
MARS Group, 2019	Low	Low	Intermediate	Low	Intermediate	Low	Low	Intermediate

**Table 4:** Detailed results of included studies.

KERRYPNX	Total ACLR	Total Re-Tears (*n* (%))	*p* Value
	(*n* = 1196)	(*n* = 73)	
McDevitt et al., 2004			>0.05
Braced	47	2 (4.3)	
Unbraced	48	3 (6.3)	
Perrone et al., 2019			0.03
Braced	135	14 (10)	
Unbraced	140	29 (21)	
MARS Group, 2019			0.23
Braced	253	5 (2)	
Unbraced	573	20 (3.5)	

**Table 5:** Summary of individual study characteristics.

Study	LOE	Dependent Variable	No of Patients (Males/Females)	Mean Age in Year (Range)	Reconstruction Technique	Follow Up	Outcomes Measured
McDevitt et al., 2004	II	Clinical and functional outcomes	Brace: 47 No brace: 48	NR: military personnel	BPTB autograph	Minimum 2 years	Subjective: IKDC, Lysholm Objective: Lachman, pivot shift, IKDC, functionl tests
Perrone et al., 2019	II	Risk for future knee injury	Brace: 135 (31/104) No brace: 140 (86/54)	Brace: 15.8 ± 1.5 No brace: 17.2 ± 1.8	Hamstring autograft	Minimum 3 years	Subjective: Marx activity score Objective: pivot shift, Lachman, MRI
MARS Group, 2019	III	Clinical and functional outcomes	Brace: 253 No Brace: 590 (482/361)	28.9 ± 10.5	BPTB/Hamstring Autograph/Allograft	2 years	Subjective: KOOS, IKDC, Marx activity scores Objective: subsequent ipsilateral knee surgery

## Data Availability

Data sharing is not applicable to this article as no new data were created or analyzed in this study.

## Appendix A. Search Strategies for the Systematic Review

**Table A1:** PubMed 9 June 2020.

#1	ACL	Anterior cruciate ligament[Mh] OR Anterior Cruciate Ligament Injuries[Mh] OR “Anterior cruciate ligament*”[tiab] OR “Anterior cruciate knee ligament*”[tiab] OR “ACL”[tiab] OR “cranial cruciate ligament*”[tiab] OR “Anterior cruciate ligament*”[OT] OR “Anterior cruciate knee ligament*”[OT] OR “ACL”[OT] OR “cranial cruciate ligament*”[OT]
#2	Reconstruction	Surgery[sh] OR “Graft*”[tiab] OR “Allograft*”[tiab] OR “Autograft*”[tiab] OR “Reconstruct*”[tiab] OR “repair*”[tiab] OR “Surger*”[tiab] OR “Graft*”[OT] OR “Allograft*”[OT] OR “Autograft*”[OT] OR “Reconstruct*”[OT] OR “Surger*”[OT] OR “repair*”[OT]
#3	Reconstruction ACL	Anterior Cruciate Ligament Reconstruction[Mh]
#4	Orthotic	Braces[mh] OR Orthotic Devices[Mh:noexp] OR “Brace*”[tiab] OR “Bracing”[tiab] OR “Orthos*”[tiab] OR “Orthes*”[tiab] OR “Orthotic”[tiab] OR “Brace*”[OT] OR “Bracing”[OT] OR “Orthos*”[OT] OR “Orthes*”[OT] OR “Orthotic”[OT]
#5	Second rupture	“Reinjur*”[tiab] OR “Re-injur*”[tiab] OR “Rerupture*”[tiab] OR “Re-rupture*”[tiab] OR “retear*”[tiab] OR “re-tear*”[tiab] OR “Graft failure*”[tiab] OR “Allograft failure*”[tiab] OR “Autograft failure*”[tiab] OR “Graft tear*”[tiab] OR “Allograft tear*”[tiab] OR “Autograft tear*”[tiab] OR “Graft injur*”[tiab] OR “Allograft injur*”[tiab] OR “Autograft injur*”[tiab] OR “Graft rupture*”[tiab] OR “Allograft rupture*”[tiab] OR “Autograft rupture*”[tiab] OR ((“Second*”[tiab] OR “contralateral”[tiab]) AND (“injur*”[tiab] OR “rupture*”[tiab] OR “tear*”[tiab])) OR “Reinjur*”[OT] OR “Re-injur*”[OT] OR “Rerupture*”[OT] OR “Re-rupture*”[OT] OR “retear*”[OT] OR “re-tear*”[OT] OR “Graft failure*”[OT] OR “Allograft failure*”[OT] OR “Autograft failure*”[OT] OR “Graft tear*”[OT] OR “Allograft tear*”[OT] OR “Autograft tear*”[OT] OR “Graft injur*”[OT] OR “Allograft injur*”[OT] OR “Autograft injur*”[OT] OR “Graft rupture*”[OT] OR “Allograft rupture*”[OT] OR “Autograft rupture*”[OT] OR ((“Second*”[OT] OR “contralateral”[OT]) AND (“injur*”[OT] OR “rupture*”[OT] OR “tear*”[OT]))
#6	Combination and limitations	((#1 AND #2) OR #3) AND #4 AND #5 AND (English[LA] OR French[LA]) 56 results

* represents any group of characters, including no character.

**Table A2:** Medline (Ovid) 9 June 2020.

1	ACL	Anterior cruciate ligament/OR Anterior Cruciate Ligament Injuries/OR (Anterior cruciate ligament* OR Anterior cruciate knee ligament* OR ACL OR cranial cruciate ligament*).ti,ab,kw,kf
2	Reconstruction	su.fs OR (Graft* OR Allograft* OR Autograft* OR Reconstruct* OR repair* OR Surger*).ti,ab,kw,kf
3	Reconstruction ACL	Exp Anterior Cruciate Ligament Reconstruction/
4	Orthotics	Braces/ OR Orthotic Devices/ OR (Brace* OR Bracing OR Orthos* OR Orthes* OR Orthotic).ti,ab,kw,kf
5	Second rupture	(Reinjur* OR Re-injur* OR Rerupture* OR Re-rupture* OR retear* OR re-tear* OR Graft failure* OR Allograft failure* OR Autograft failure* OR Graft tear* OR Allograft tear* OR Autograft tear* OR Graft injur* OR Allograft injur* OR Autograft injur* OR Graft rupture* OR Allograft rupture* OR Autograft rupture* OR ((Second* OR contralateral) AND (injur* OR rupture* OR tear*))).ti,ab,kw,kf
6	Combination and limitations	((1 AND 2) OR 3) AND 4 AND 5 AND (English OR French).lg 55 results

* represents any group of characters, including no character.

**Table A3:** All EBM Reviews (Ovid) 9 June 2020.

1	ACL	Anterior cruciate ligament/OR Anterior Cruciate Ligament Injuries/OR (Anterior cruciate ligament* OR Anterior cruciate knee ligament* OR ACL OR cranial cruciate ligament*).ti,ab,kw,kf
2	Reconstruction	su.fs OR (Graft* OR Allograft* OR Autograft* OR Reconstruct* OR repair* OR Surger*).ti,ab,kw,kf
3	Reconstruction ACL	Exp Anterior Cruciate Ligament Reconstruction/
4	Orthotics	Braces/OR Orthotic Devices/OR (Brace* OR Bracing OR Orthos* OR Orthes* OR Orthotic).ti,ab,kw,kf
5	Second rupture	(Reinjur* OR Re-injur* OR Rerupture* OR Re-rupture* OR retear* OR re-tear* OR Graft failure* OR Allograft failure* OR Autograft failure* OR Graft tear* OR Allograft tear* OR Autograft tear* OR Graft injur* OR Allograft injur* OR Autograft injur* OR Graft rupture* OR Allograft rupture* OR Autograft rupture* OR ((Second* OR contralateral) AND (injur* OR rupture* OR tear*))).ti,ab,kw,kf
6	Combination and limitations	((1 AND 2) OR 3) AND 4 AND 5 AND (English OR French).lg 9 results

* represents any group of characters, including no character.

**Table A4:** Embase (Ovid) 9 June 2020.

1	ACL	Anterior cruciate ligament/OR exp Anterior Cruciate Ligament Injury/OR (Anterior cruciate ligament* OR Anterior cruciate knee ligament* OR ACL OR cranial cruciate ligament*).ti,ab,kw
2	Reconstruction	su.fs OR (Graft* OR Allograft* OR Autograft* OR Reconstruct* OR repair* OR Surger*).ti,ab,kw
3	Reconstruction ACL	Anterior Cruciate Ligament Reconstruction/
4	Orthotics	Exp knee orthosis/OR orthosis/OR brace/OR (Brace* OR Bracing OR Orthos* OR Orthes* OR Orthotic).ti,ab,kw
5	Second rupture	(Reinjur* OR Re-injur* OR Rerupture* OR Re-rupture* OR retear* OR re-tear* OR Graft failure* OR Allograft failure* OR Autograft failure* OR Graft tear* OR Allograft tear* OR Autograft tear* OR Graft injur* OR Allograft injur* OR Autograft injur* OR Graft rupture* OR Allograft rupture* OR Autograft rupture* OR ((Second* OR contralateral) AND (injur* OR rupture* OR tear*))).ti,ab,kw
6	Combination and limitations	((1 AND 2) OR 3) AND 4 AND 5 AND (English OR French).lg 102 results

* represents any group of characters, including no character.

**Table A5:** Sportdiscus EBSCO 9 June 2020.

S1	ACL	DE(Anterior cruciate ligament) OR DE(Anterior Cruciate Ligament Injuries) OR TI(Anterior cruciate ligament* OR Anterior cruciate knee ligament* OR ACL OR cranial cruciate ligament*) OR AB(Anterior cruciate ligament* OR Anterior cruciate knee ligament* OR ACL OR cranial cruciate ligament*) OR KW(Anterior cruciate ligament* OR Anterior cruciate knee ligament* OR ACL OR cranial cruciate ligament*)
S2	Reconstruction	TI(Graft* OR Allograft* OR Autograft* OR Reconstruct* OR repair* OR Surger*) OR AB(Graft* OR Allograft* OR Autograft* OR Reconstruct* OR repair* OR Surger*) OR KW(Graft* OR Allograft* OR Autograft* OR Reconstruct* OR repair* OR Surger*)
S3	Reconstruction ACL	DE(Anterior cruciate ligament surgery)
S4	Orthotics	DE(ORTHOPEDIC braces) OR DE(KNEE braces) OR TI(Brace* OR Bracing OR Orthos* OR Orthes* OR Orthotic) OR AB(Brace* OR Bracing OR Orthos* OR Orthes* OR Orthotic) OR KW(Brace* OR Bracing OR Orthos* OR Orthes* OR Orthotic)
S5	Second rupture	TI(Reinjur* OR Re-injur* OR Rerupture* OR Re-rupture* OR retear* OR re-tear* OR Graft failure* OR Allograft failure* OR Autograft failure* OR Graft tear* OR Allograft tear* OR Autograft tear* OR Graft injur* OR Allograft injur* OR Autograft injur* OR Graft rupture* OR Allograft rupture* OR Autograft rupture* OR ((Second* OR contralateral) AND (injur* OR rupture* OR tear*))) OR AB(Reinjur* OR Re-injur* OR Rerupture* OR Re-rupture* OR retear* OR re-tear* OR Graft failure* OR Allograft failure* OR Autograft failure* OR Graft tear* OR Allograft tear* OR Autograft tear* OR Graft injur* OR Allograft injur* OR Autograft injur* OR Graft rupture* OR Allograft rupture* OR Autograft rupture* OR ((Second* OR contralateral) AND (injur* OR rupture* OR tear*))) OR KW(Reinjur* OR Re-injur* OR Rerupture* OR Re-rupture* OR retear* OR re-tear* OR Graft failure* OR Allograft failure* OR Autograft failure* OR Graft tear* OR Allograft tear* OR Autograft tear* OR Graft injur* OR Allograft injur* OR Autograft injur* OR Graft rupture* OR Allograft rupture* OR Autograft rupture* OR ((Second* OR contralateral) AND (injur* OR rupture* OR tear*)))
S6	Combination and limitations	((S1 AND S2) OR S3) AND S4 AND S5 AND LA(English OR French) 27 results

* represents any group of characters, including no character.

**Table A6:** ISI Web of Science 9 June 2020.

#1	ACL	TS = (Anterior cruciate ligament* OR Anterior cruciate knee ligament* OR ACL OR cranial cruciate ligament*)
#2	Reconstruction	TS = (Graft* OR Allograft* OR Autograft* OR Reconstruct* OR repair* OR Surger*)
#3	Orthotics	TS = (Brace* OR Bracing OR Orthos* OR Orthes* OR Orthotic)
#4	Second rupture	TS = (Reinjur* OR Re-injur* OR Rerupture* OR Re-rupture* OR retear* OR re-tear* OR Graft failure* OR Allograft failure* OR Autograft failure* OR Graft tear* OR Allograft tear* OR Autograft tear* OR Graft injur* OR Allograft injur* OR Autograft injur* OR Graft rupture* OR Allograft rupture* OR Autograft rupture* OR ((Second* OR contralateral) AND (injur* OR rupture* OR tear*)))
#5	Combination and limitations	#1 AND #2 AND #3 AND #4 AND LANGUAGE:(English OR French) 104 results

* represents any group of characters, including no character.
